# Quality and extent of implementation of a nurse-led care management intervention: care coordination for health promotion and activities in Parkinson’s disease (CHAPS)

**DOI:** 10.1186/s12913-020-05594-8

**Published:** 2020-08-10

**Authors:** Karen I. Connor, Hilary C. Siebens, Brian S. Mittman, David A. Ganz, Frances Barry, E. J. Ernst, Lisa K. Edwards, Michael G. McGowan, Donna K. McNeese-Smith, Eric M. Cheng, Barbara G. Vickrey

**Affiliations:** 1 Veterans Affairs Southwest Parkinson’s Disease Research, Education and Clinical Center, Los Angeles, CA USA; 2 University of California, Los Angeles David Geffen School of Medicine, Los Angeles, CA USA; 3 Novato, USA; 4 Siebens Patient Care Communications LLC, Seal Beach, CA USA; 5 Kaiser Permanente Research, Pasadena, CA USA; 6 Veterans Affairs Geriatric Research, Education and Clinical Center and Center for the Study of Healthcare Innovation, Implementation and Policy, Los Angeles, CA USA; 7 American Association of Nurse Practitioners, Austin, TX USA; 8 UCLA School of Nursing, Los Angeles, CA USA; 9 Icahn School of Medicine at Mount Sinai, New York, NY USA

**Keywords:** Parkinson disease, Patient care management, Health services, Nursing process, Health communication, Case manager

## Abstract

**Background:**

A recent nurse-led, telephone-administered 18-month intervention, Care Coordination for Health Promotion and Activities in Parkinson’s Disease (CHAPS), was tested in a randomized controlled trial and improved care quality. Therefore, intervention details on nurse care manager activity (types and frequencies) and participant actions are needed to support potential dissemination. Activities include nurse care manager use of a holistic organizing framework, identification of Parkinson's disease (PD)-related problems/topics, communication with PD specialists and care coordination, participant coaching, and participant self-care actions including use of a notebook self-care tool.

**Methods:**

This article reports descriptive data on the CHAPS intervention. The study setting was five sites in the Veterans Affairs Healthcare System. Sociodemographic data were gathered from surveys of study participants (community-dwelling veterans with PD). Nurse care manager intervention activities were abstracted from electronic medical records and logbooks. Statistical analysis software was used to provide summary statistics; closed card sorting was used to group some data.

**Results:**

Intervention participants (*n* = 140) were primarily men, mean age 69.4 years (standard deviation 10.3) and community-dwelling. All received the CHAPS Initial Assessment, which had algorithms designed to identify 31 unique CHAPS standard problems/topics. These were frequently documented (*n* = 4938), and 98.6% were grouped by assigned domain from the Organizing Framework (Siebens Domain Management Model™). Nurse care managers performed 27 unique activity types to address identified problems, collaborating with participants and PD specialists. The two most frequent unique activities were counseling/emotional support (*n* = 387) and medication management (*n* = 349). Both were among 2749 total performed activities in the category Implementing Interventions (coaching). Participants reported unique self-care action types (*n* = 23) including use of a new notebook self-care tool.

**Conclusions:**

CHAPS nurse care managers implemented multiple activities including participant coaching and care coordination per the CHAPS protocol. Participants reported various self-care actions including use of a personalized notebook. These findings indicate good quality and extent of implementation, contribute to ensuring reproducibility, and support CHAPS dissemination as a real-world approach to improve care quality.

**Trial registration:**

ClinicalTrials.gov as NCT01532986, registered on January 13, 2012.

## Background

Parkinson disease (PD) is a progressive and enduring (chronic) condition encompassing a wide range of symptoms, signs, and associated problems [1] including tremors and rigidity with slowness in movement. As the disease progresses, additional distressing symptoms occur that also affect health and well-being (e.g., sleep and fatigue, dementia, depression, falls). Innovative care management approaches, that are person/patient-centered such as assessment-driven health coaching and care coordination, can support patients and family caregivers in self-care [2–9]. For PD, studies have highlighted the role of nurses in providing and coordinating this care [10–16], given patient needs for added support [17]. Some experts and payors have proposed that an experienced PD nurse, as a care manager, could ensure individualized, appropriate, and integrated care [18] and guidelines for PD nurse specialists have been presented [19]. Despite promising evidence, nurse care management remains under-utilized for PD, likely due to common challenges confronting clinicians and organizations [20]. Yet interventions that are explicit, reproduceable, and acceptable to stakeholders offer considerable potential to help decrease practice variation and improve care for enduring health conditions. Thus, the quality and extent of a PD intervention’s implementation need to be described and compared to this literature to aid in decisions about its dissemination [21].

In a previous article, we reported results of a randomized trial of a nurse-led proactive telephone-based, 18-month PD care management intervention, Care Coordination and Health Promotion and Activities in Parkinson’s Disease (CHAPS), across five Veterans Health Administration (VA) medical centers in the southwest United States [22]. VA healthcare facilities provide primary and specialty medical care with social service supports for men and women who have served in the United States military. Study participants (veterans) were living in the community and had lower health-related quality of life [22] compared to other community-dwelling adults with PD [23], yet like veterans in another VA study [24]. The CHAPS usual care arm participants received health care through PD specialists with limited nursing support.

Trial outcomes demonstrated improvements in PD care quality as measured by greater adherence to a set of 18 PD quality care indicators developed by an expert panel [25, 26] that were likely to be affected by nurse care management. These ranged from assessment and counseling on PD medication side effects, management of non-motor complications of PD, to non-pharmacologic approaches to PD management. Among secondary outcomes, a statistically significant improvement was seen in depressive symptomatology, which was lower in the intervention arm vs. usual care as measured by the Patient Health Questionnaire-2 screening tool [22, 27]. These findings support CHAPS dissemination; however, additional prerequisites are needed. The aim of this study is to examine the quality (activities that occurred) and extent (frequency of these activities) during implementation of the CHAPS protocol in a real-world setting [21]. These data may add support for CHAPS’ dissemination into routine clinical care [28, 29], contribute to ensuring CHAPS reproducibility, and aid iterative research in PD care.

### The intervention

**Fig. 1:**
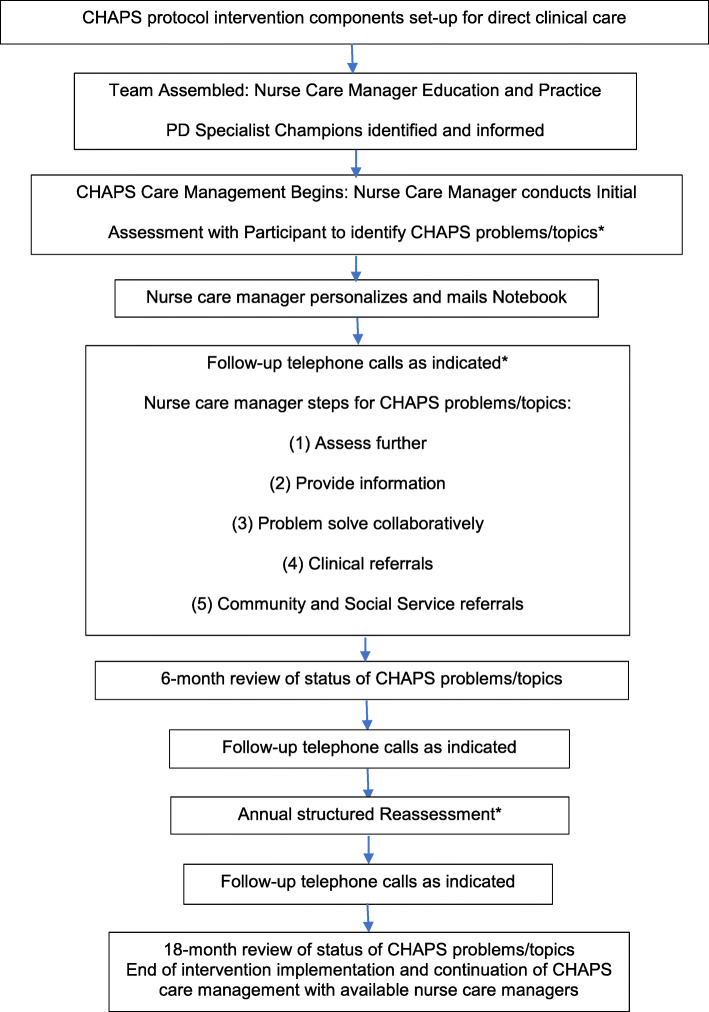
The CHAPS Nurse Care Manager - Telephone-administered Intervention. Decision-support ongoing in (1) monthly huddles between nurse care managers and Parkinson disease specialist champions and (2) nurse care manager biweekly conference call. * Average time spent on the telephone with participants for the Assessment was 120 SD 78 min; for follow-up calls 28 SD 20 min, and for Reassessment 34 SD 32 min. See Connor KI, Cheng EM, Barry F, Siebens HC, Lee ML, Ganz DA, Mittman BS, Connor MK, Edwards LK, McGowan, Vickrey BG. Randomized Trial of Care Management to Improve Parkinson Disease Care Quality. Neurology. 2019;92: e1831-e1842. SD – standard deviation

As described in detail in the study protocol [30], the CHAPS program was based on the Chronic Care Model [31] and designed to operationalize and facilitate adherence to care recommendations derived from PD practice guidelines and clinical expertise. The intervention sequence of activities with participants is summarized in Fig. 1. A ratio of 1:135 full time employee equivalent (FTEE) nurse care manager to veterans with PD (participants) was planned, based on clinical experience with veterans as motivated self-managers of their PD and the researchers’ previous experience with dyads of patients with dementia and their caregivers (1:100 individuals) [32].

The Siebens Domain Management Model™ [33] provided an organizing framework [34] to facilitate application of a practical person-centered perspective and to design of a structured CHAPS Assessment containing 31 CHAPS standard problem/topic types, plus guidance for follow-up encounters and the design of My Action Plan (a personalized plan of action) [30]. The four domains were: I Medical/Surgical Issues, II Mental Status/Emotions/Coping, III Physical Function, and IV Living Environment (© Hilary C Siebens MD 2005). This interdisciplinary, evidence-based Organizing Framework, proposed for geriatric assessment in the community [35], has been applied to emergency department care for high risk older adults [36, 37], physician documentation [38], and inpatient rehabilitation team conferences yielding improved patient outcomes [39–41].

The CHAPS Assessment, administered by the nurse care manager, was comprised of questions to identify participant strengths (e.g., taking medication correctly or a pertinent negative such as no trouble walking) and problems/topics using structured questions. Validated instruments such as the Epworth Sleep Scale and Patient Healthcare Questionnaire-9 were included. Embedded algorithms triggered problems/topics needing to be addressed [30]. Each problem/topic was assigned to one of the Organizing Framework’s four domains (Table 1). The CHAPS Assessment also contained questions about learning mode preferences and tracking of health information [42].

**Table 1:** Framework to organize Parkinson disease health-related problems/topics into 4-domains^a^

Domains	Sub-domains	Corresponding CHAPS Standard Problems/Topics
I Medical/Surgical Issues		
	Diseases	
		2 Medication
	Symptoms	3 Motor-related
		4 Gastrointestinal-related
		5 Weight/Nutrition/Dental
		6 Swallowing
		7 Urology-related
		8 Pain
		9 Sleep and Fatigue
	Prevention	1 Prevention
		Other problem/topic
II Mental Status/Emotions/Coping		
	Communication	10 Hearing
		11 Vision
		12 Speech
	Cognition	13 Cognition
		14 Psychosis/Hallucinations
	Emotions	15 Depressive symptomatology
		16 Anxiety
	Coping/Behaviors	17 Understanding Parkinson’s disease
		18 Coping/Self-management
		19 Apathy
		20 Impulse Control Disorder
	Spirituality	Single direct question in Assessment
	Patient Preferences	21 Preferences/Long term care planning
		Other problem/topic
III Physical Function		
	Basic ADL (BADL)	22 Functional Limitations
		23 Falls (in the home)
	Intermediate ADL (IADL)	22 Functional Limitations
		23 Falls (outside the home)
	Advanced ADL (AADL)	24 Physical Activity
		25 Driving
		Other problem/topic
IV Living Environment		
	A. Physical Environment	A. Physical Environment
	B. Social Environment	B. Social Environment
		26 Elder Abuse
	C. Financial/Community Resources	C. Financial/Community Resources
		27 Access to care
		28 End of Life Resources
		Other problem/topic

*CHAPS* Care Coordination for Health Promotion and Activities in Parkinson’s Disease

^a^ Four domains and their respective sub-domain headings from the Siebens Domain Management Model™ (SDMM™) (Organizing Framework) used with permission. ADL – Activities of daily living

CHAPS included two self-management tools: My HealtheVet (the VA’s electronic patient portal) and the Siebens Health Care Notebook (Notebook) with four sections based on the Organizing Framework using plain domain name phrases developed with input from health literacy experts [30, 43–45]. For participant information and coaching, each section included an education sheet: Levodopa and Interactions with Protein (I), PD at Home (monthly free VA supportive and educational conference call) (II), Exercise and Parkinson disease (III), and Fall Proofing Your Home (IV). All Notebooks were personalized by adding nurse care manager contact information and a photograph, the CHAPS Assessment, and My Action Plan. The Notebook served as a repository of patient reminders [17], such as after visit summaries, education sheets, and health-tracking logs, to assist in information management and communication [42] and to aid in any care transitions [46] if they occurred.

CHAPS problem/topic intervention protocols were available to nurse care managers as guides for problem solving and care delivery. These were organized using a framework of intervention protocol steps, each with care recommendations: (1) assess further, (2) provide information, (3) problem-solve collaboratively, (4) discuss/facilitate clinical referrals, and (5) discuss/facilitate community and social service referrals (Table 2) [30]. These plans were adapted from a care management intervention for dementia [32]. These steps were consistent with motivational interviewing that includes asking, listening, and informing [47, 48] and self-management skill building [49]. To provide information for use in coaching, 84 unique problem-specific education sheets were gathered from VA Parkinson’s Disease Research, Education, and Clinical Centers (PADRECC) and other organizations. Nurse care managers documented their CHAPS work in the VA electronic medical record.

**Table 2:** Example of a CHAPS Problem/Topic Intervention Protocol

MEDICATIONS	
Nurse care manager Steps for CHAPS Problems/Topics	Examples
Assess further^a^	- What is your routine for taking medications – what times, with meals? - How do you remember to take your medications (alarm, watch, clock, varies – a red flag)? etc. - NOTE: Compare to electronic medical record?
Provide information	- Educate that a routine for mediation-taking is important (put dose next to toothbrush, etc.) “Same time every time” - Recommend taking medications when “on”. - Use Siebens Health Care Notebook to provide information. Review levodopa-protein interaction education sheet at end of Section 1, etc.
Problem solve collaboratively	- My HealtheVet - encourage use of prescription refills. - Offer options to use Notebook to help self-manage medication issues (1) Use it to maintain a current medication list in Section 1; (2) If hospitalized, show hospital physicians Provider’s Quick Fact Sheet in Section 6, etc. - Evaluate collaboratively and coach on strategies based on the cause(s): (1) If forgetfulness, then alarm devices (like smart cell phone alarm?); (2) If cause is complexity, then organizers. etc.
Clinical referrals – discuss/facilitate	- Contact Parkinson disease specialist or neurology consultant for Parkinson’s disease at your facility. - Refer to Parkinson disease specialist or neurologist to discuss dopaminergic medication (has new impairment in ADLs). - Consider referring to Pharmacy for a medication review. etc.
Community and Social Service referrals – discuss/facilitate	- Refer to Social Services for financial or other resources relevant to a medication issue. - Refer to local Veteran Service Organization (VSO). - Refer to Business Office of his or her local Veteran Affairs Medical Center. etc.

^a^ Assess further is root cause analysis of the problem/topic at the individual participant level

## Methods

Study aims were to describe: (1) type and frequency of PD problems/topics (2) use of the 4-domain Organizing Framework, (3) type and frequency of nurse care manager activities, and (4) evidence of participant self-care (participant actions and use of the Notebook).

### Setting and eligible participants

**Fig. 2:**
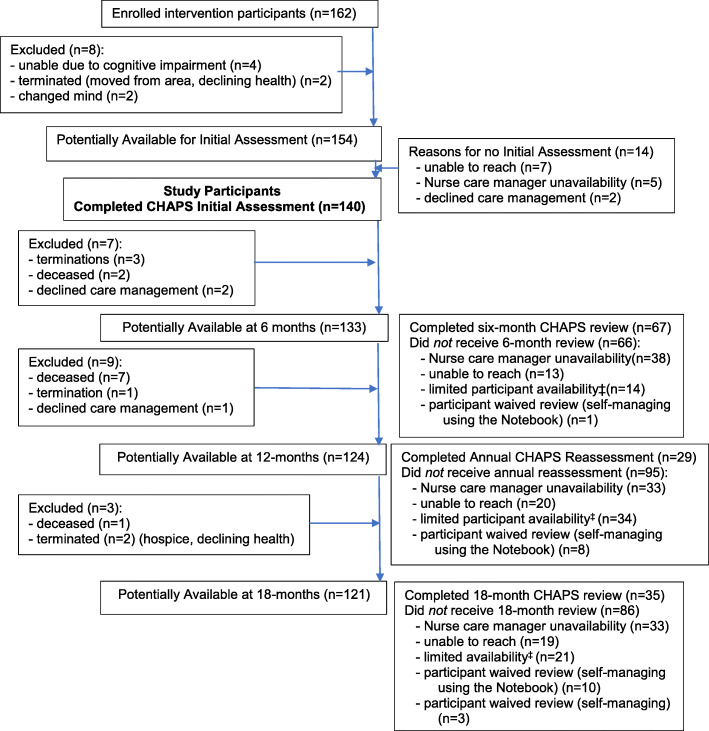
Flow diagram of participants availability and receipt of CHAPS nurse care manager assessments and reviews. ^‡^ limited availability (e.g. medical, coping, family, financial issues)

Details of the five participating VA medical centers in the southwest United States and participant eligibility have been published [22]. Participants in this report were a subset, *n* = 140, of the 162 participants assigned to the intervention arm who received, at minimum, the CHAPS initial Assessment (Fig. 2).

### Data

#### Baseline participant characteristics

Socio-demographics and a utility measure of health status/health-related quality of life (Health Utilities Index3) [50] were collected via baseline telephone survey by a study research assistant [22]. Other baseline variables were abstracted from CHAPS Assessment notes documented by the nurse care managers, including preferred methods of learning information, what and where information was kept, and use of the Internet and My HealtheVet, and use of providers and health insurance outside the VA.

#### CHAPS problems/topics and care management activities

The research assistant and project manager used the electronic medical record nurse care manager documentation abstraction tool developed by the research team and modified from prior work [51]. They abstracted note type, mode of communication with participant, who initiated the communication, and who participated on the telephone call. They abstracted nurse care manager-identified problems/topics using a list of CHAPS problems/topics, sub-divided, for ease of abstraction, into the four domains of the Organizing Framework.

The abstractors collected information on nurse care manager activities, using a list adapted from a prior care management study [52]. These data were recorded in a Research Electronic Data Capture (REDCap) database. Care coordination activities were measured three ways: (1) nurse care manager referrals to providers and services; (2) nurse care manager recommendations on topics participants could discuss at referral appointments; and (3) warm-hand off methods: (a) how communication was done (live concurrent phone discussion, provider co-signature of nurse care manager note, face-to-face discussions, and secure email) and (b) with whom (e.g., medical disciplines, care partner). Details about Notebook coaching by nurse care managers were also abstracted. Mailing of the participants’ Notebooks and problem-specific education sheets were tracked in electronic logbooks. If care management ended prior to the 18-month intervention, reasons were recorded in the study’s REDCap database.

#### Participants’ concerns, self-care actions, and improvements

The abstractors pulled data from nurse care manager CHAPS notes on problems/topics that participants identified as concerns. They abstracted participants’ self-care actions using a list adapted from previous work on dementia care management (unpublished, KIC). This list was subdivided, for ease of abstraction, into the 4-domains from the Siebens Domain Management Model (I Medical/Surgical Issues, II Mental Status/Emotions/Coping, III Physical Function, and IV Living Environment), based on problem addressed [34]. For problems/topics in which the nurse care manager documented participant reported improvement, the abstractors noted the associated CHAPS problems/topics.

#### Quality check

Two authors (KIC, EMC) created help lists (cues of acceptable text for variables) to reduce abstraction variability. Abstractors met with the principal investigator (KIC) to resolve any questions about data to be abstracted. Data were entered in REDCap. Quality of data gathering completeness was examined by determining if all notes per case were pulled by the abstractors for 25 randomly selected patients (18% of the participant sample (*n* = 140) being examined in this study).

#### Analyses

SAS 9.4 statistical analysis software (SAS Institute, Inc., Cary, North Carolina) was used to generate descriptive summary statistics (i.e., frequencies, percentages, and standard deviations). Some frequencies were reported as both total number of items (e.g. notes, problem frequency) and number of unique participants involved. For descriptive purposes, closed card sorting (predefined categories) was used to group some nurse care manager activities into predefined categories. Card sorting is a method to categorize unique items into groups [52, 53]. The statistician pulled the activity data from REDCap into an excel spread sheet. A nurse health services researcher (KIC) and physician (HCS), orally read and jointly examined the activity on each line, and then placed each item into one of the predetermined categories in another column on the excel spread sheet. For every disagreement, the categorization was discussed by the two researchers. Based on subject matter knowledge, each of these items was assigned to its respective group when agreement was reached.

## Results

### Quality check

A quality check, verifying the number of nurse care manager CHAPS notes that had been pulled for abstraction, was completed. Of the 25 cases, 24 (96%) had 100% of notes pulled and then abstracted.

### Types and frequency of nurse care manager CHAPS notes

A flow diagram (Fig. 2) showed participant trial terminations and potential availability for nurse care manager calls. A total of 722 nurse care manager CHAPS notes on these 140 participants were identified for abstraction. Of these, several types of brief notes (*n* = 66) were not included in these analyses (i.e., voicemails, change of nurse care manager, unable to reach letters, and death of a participant). Of the remaining notes (*n* = 656), 633 (96.5%) documented telephone calls and 23 (3.5%) were in person medical office visits.

After the initial assessment, the total number of nurse care manager telephone calls per participant varied from zero to six or more. A total of 95% of the calls with participants (*n* = 623) were initiated by nurse care managers and 5% (*n* = 33) were participant-initiated. Additionally, care partners participated in 129 calls (19.7%) as requested by participants.

### Participant characteristics

Characteristics of the 140 participants at baseline did not differ from the 22 participants who did not receive the intervention (Table 3). The nurse care managers, to help in their coaching, assessed participants preferred methods for learning new information. The most frequent methods were reading printed material (*n* = 84, 60.0%), one-on-one conversations (*n* = 68, 48.6%), and Internet (*n* = 61, 43.6%). Nurse care managers also identified how participants kept track of health information: the most frequent were in folders (*n* = 51, 34.6%), on calendars (*n* = 38, 27.1%), in computers (*n* = 23, 16.4%), and in cell phones (*n* = 22, 15.7%). A variety of health-related information was kept in multiple places in participant homes. Furthermore, nurse care managers identified that most participants had access to the Internet (*n* = 112, 82.9%). More than half had heard of My HealtheVet (*n* = 86, 61.4%). For those who utilized it (*n* = 45), the most frequent uses were medication refills (*n* = 29), recording medical care information (n = 23), writing secure messages (*n* = 17), looking up information on their health conditions (n = 11), and reviewing vital signs and test results (*n* = 10). Difficulty in using the computer was the most frequently cited barrier to use.

**Table 3:** Study population characteristics

	Completed initial assessment (*n* = 140)	Did not complete initial assessment (n = 22)	*p*-value
Age, years, mean (std)	69.4 (10.3)	71.2 (8.9)	0.4418
Gender, n (%)			0.2836
Male	133 (95.0)	22 (100.0)	
Female	7 (5.0)	–	
Ethnicity, n (%)			0.4601
African American	7 (5.0)	2 (9.1)	
Asian American	2 (1.4)	1 (4.5)	
First Nation or Alaskan Native	2 (1.4)	–	
Caucasian or Euro-American	107 (76.4)	18 (81.8)	
Hispanic or Latino	20 (14.3)	1 (4.5)	
Other	2 (1.4)	–	
Primary language spoken, n (%)			0.5991
English	140 (100.0)	22 (100.0)	
Education, n (%)			0.4818
More than 4-college degree	26 (18.6)	7 (31.8)	
4-year college graduate	27 (19.3)	5 (22.7)	
Some college or 2-year degree	56 (40.0)	5 (22.7)	
At least high school graduate or equivalent	19 (13.6)	4 (18.2)	
Some high school	11 (7.9)	1 (4.5)	
8th grade or less	1 (0.7)	–	
Employment, n (%)			0.7955
Working for pay full-time	5 (3.6)	1 (4.5)	
Working for pay part-time	13 (9.4)	1 (4.5)	
Working as a homemaker in my own home	–	–	
Unemployed but looking for work	4 (2.9)	–	
Cannot work because of health disability	35 (25.2)	4 (18.2)	
Retired	82 (59.0)	16 (72.7)	
Health Utilities Index (HUI3), mean (std)*(Range: −0.36 (worst) to 1 (best))*	0.45 (0.31)	0.34 (0.23)	0.1305

*std* standard deviation

During the 6 months prior to CHAPS, the nurse care managers assessed veteran access to care and determined that more than half of the participants (*n* = 73, 52.1%) reported seeing providers outside the VA. These were, most frequently, primary care physicians, PD specialists, and other specialists (e.g., dentists, urologists, psychiatrists). These participants utilized non-VA health insurance plans and cash to pay for these services.

### Preparing the CHAPS clinical team

The research team identified a PD specialist champion (i.e., a project advocate) at each site. The principal investigator oriented them and site staff to CHAPS and provided a sample 3-ring participant Notebook with examples of printed education sheets for participants. Modes of decision-support were discussed: (1) clinical huddles between nurse care managers and PD specialist champions and (2) CHAPS care management conference calls among nurse care managers.

The nurse care managers (*n* = 8) had varying nursing degrees: 2 doctorates, 4 masters, 1 bachelor’s, 1 associate degree. Some had additional licenses/credentials: 3 nurse practitioners, 1 advance practice nurse, and 1 had expertise in PD care. Nurse care managers were hired for part-time positions ranging from 4 to 20 h per week. These positions were paid by the national PADRECC rather than through research funds. On average, nurse care managers had a patient-panel size of approximately 1:125 nurse care manager FTEE to participants with PD, slightly less than 1:135 that was planned.

Two authors (KIC, HCS) conducted orientation to CHAPS care management through a one-on-one structured 10 to 40-h program, depending on the nurse’s background via telephone or in person. Introductory readings covered military cultural competence, managing complexities of PD, review of *Davis Phinney Every Victory Counts* [54], and the 3-ringed CHAPS Nurse Care Manager Binder.

The binder, also available in electronic format, contained: (1) the Organizing Framework; (2) directions for implementing each step in CHAPS; (3) templates for various tools including the Assessment Summary, My Action Plan, and Follow-up Notes, and (4) a guide for pre-populating relevant data from the electronic medical record (e.g., most recent flu shot) into the CHAPS Assessment before telephoning the participant. Additionally, there were: (1) a user manual for the CHAPS Assessment Microsoft Access database; (2) a guideline on Notebook use; (3) intervention protocol recommendations for each CHAPS problem/topic; (4) patient-panel tracking tool user guide; and (5) protocols for clinical huddles, suicide risk, serious adverse events reporting, and sending caregiver packets for education and psychosocial (i.e., emotional) support. A national/regional/local community resource list was compiled by the nurse care managers and available electronically. Research team members (KIC, EMC, HCS, LKE) provided hands-on practice with the above tools. Nurses became CHAPS nurse care managers after completing all CHAPS orientation activities using a 16-item check-off list.

### Types and frequency of CHAPS problems/topics

The nurse care managers identified 5201 problems/topics as documented in 656 CHAPS Assessment and follow-up notes. Of these, 4938 covered the 31 unique CHAPS problem/topic types while the remaining 263 were other additional problems/topics (Table 4).

**Table 4:** Types and Frequency of CHAPS problems addressed by nurse care managers in notes and by unique participant

Problems/Topics	Notes (n = 656) n (%)	Participants (n = 140) n (%)
I Medical/Surgical Issues^a^		
1 Prevention	219 (33.4)	100 (71.4)
2 Medication	349 (53.2)	125 (89.3)
3 Motor-related	302 (46.2)	104 (74.3)
4 Gastrointestinal-related	187 (28.5)	85 (60.7)
5 Weight/Nutrition/Dental	208 (31.9)	89 (63.6)
6 Swallowing	118 (18.0)	49 (35.0)
7 Urology-related	155 (23.8)	86 (61.4)
8 Pain	189 (29.0)	87 (62.1)
9 Sleep and Fatigue	179 (27.4)	84 (60.0)
II Mental Status/Emotions/Coping		
10 Hearing	82 (12.5)	38 (27.1)
11 Vision	98 (14.9)	47 (33.6)
12 Speech	113 (17.2)	54 (38.6)
13 Cognition	112 (17.1)	50 (35.7)
14 Psychosis/Hallucinations	36 (5.5)	24 (17.1)
15 Depressive symptomatology	175 (26.7)	65 (46.4)
16 Anxiety	96 (14.6)	37 (26.4)
17 Understanding Parkinson’s disease	244 (37.2)	106 (75.7)
18 Coping/Self-management	192 (29.3)	74 (52.9)
19 Apathy	40 (6.1)	22 (15.7)
20 Impulse Control Disorder	32 (4.9)	15 (10.7)
21 Preferences/Long term care planning	144 (22.0)	74 (52.9)
III Physical Function		
22 Functional Limitations	188 (28.7)	79 (56.4)
23 Falls	272 (41.5)	106 (75.7)
24 Physical Activity	154 (23.5)	64 (45.7)
25 Driving	94 (14.3)	49 (35.0)
IV Living Environment		
A. Physical Environment	266 (40.5)	98 (70.0)
B. Social Environment	274 (41.8)	100 (71.4)
26 Elder Abuse	1 (0.2)	1 (0.7)
C. Financial and Community Resources	258 (39.3)	99 (70.7)
27 Access to Care	137 (20.9)	59 (42.1)
28 End of Life Resources	24 (3.7)	12 (8.6)

^a^ Headings are from the Siebens Domain Management Model™, used with permission

### Use of the organizing framework in nurse care manager documentation

Nurse care managers used the Organizing Framework domain headings per protocol in documentation for 4870 of 5201 (97.7%) of the CHAPS problems/topics. These were distributed over the 4 domains: I Medical/Surgical Issues 38.8%, II Mental Status/Emotions/Coping 27.5%, III Physical Function 14.3%, and Living Environment 19.4%.

### Nurse care manager activities

All of the 27 nurse care manager activity types were closed card sorted into five nursing process categories [55, 56]: Nursing assessments, Nursing diagnoses, Planning outcomes, Implementing interventions (i.e., coaching in outpatient proactive care management), Evaluating, and Other. The nurse care managers most frequently provided counseling/emotional support. Other frequent activities were: (1) discussed medication management, (2) provided verbal education, (3) initiated care coordination, (4) discussed/made referrals, and (5) discussed the Siebens Health Care Notebook (Table 5).

**Table 5:** Frequency of nurse care manager activities in notes and by unique number of participants

Nurse Care Management Activities	Notes	Participants
	(n = 656)	(n = 140)
	n (%)	n (%)
Nursing Assessment		
Administered initial or re-assessment	169 (25.8) ^a^	140 (100.0)
Reviewed transitional care after a hospitalization	11 (1.6)	9 (6.4)
Nursing Diagnoses		
Identified/discussed new problem(s)	267 (39.6)	112 (80.0)
Algorithm-identified problems in initial or reassessment	169 (25.8)	140 (100.0)
Planning Outcomes (Goal Setting)		
Motivational collaborative problem-solving	144 (21.4)	71 (50.7)
Implementing Interventions (Coaching)		
Provided counseling + emotional support	387 (57.4)	121 (86.4)
Discussed medication management	349 (51.8)	124 (88.6)
Provided education – verbal	310 (46.0)	107 (76.4)
Initiation of care coordination	289 (42.9)	95 (67.9)
Discussed/made referrals	279 (41.4)	98 (70.0)
Discussed Siebens Health Care Notebook	222 (32.9)	108 (77.1)
Encouraged/assisted in making appointments	173 (25.7)	79 (56.4)
Provided education – written materials to be mailed	168 (24.9)	102 (72.9)
Facilitation of social support	140 (20.8)	63 (45.0)
Recommended topic/intervention to discuss with a provider	129 (19.1)	61 (43.6)
Encouraged follow-through with a provider	103 (15.3)	51 (36.4)
Recommended a specific care intervention to participant	74 (11.0)	45 (32.1)
Provided education online or electronic (DVD) resources	68 (10.1)	38 (27.1)
Discussed Davis Phinney Binder	30 (4.5)	18 (12.9)
Discussed My Action Plan	13 (1.9)	11 (7.9)
Recommended voice exercises	8 (1.2)	5 (3.6)
Discussed stress management	7 (1.0)	7 (5.0)
Evaluating		
Followed-up to monitor progress/ follow-up on prior problems	375 (55.6)	113 (80.7)
Followed-up involving care coordination/prior referrals	93 (13.8)	39 (27.9)
Formal discussion of initial assessment	16 (2.4)	15 (10.7)
Formal discussion of reassessment	1 (0.1)	1 (0.7)
Other	18 (2.7)	15 (10.7)
Documented future nurse care manager plans	465 (69.0)	119 (85.0)

^a^ This represents the 140 initial assessments and the 29 reassessments

*DVD* digital versatile disc

All data from two of these 27 activities were further closed card sorted to examine specifics on nurse care manager coaching. One activity was the “Recommended topics/interventions for participant to discuss with their providers”. The second activity was “Recommended a specific care intervention to participant”.

Of the activity, “Recommended topic/intervention to discuss with a provider” (*n* = 129) (Table 5), a total of 155 specific suggestions were available and abstracted. These were closed card sorted into the 31 CHAPS problems/topics. The most frequent among each domain were: (I) Medications (*n* = 27) (e.g., discuss medication duplication between VA and outside providers) and Other medical/surgical issues (*n* = 18) (e.g., discuss dizziness and blood pressure readings with neurologist); (II) Vision (*n* = 5) (e.g., talk with primary care physician about vision screening) and Coping/Self-management (n = 5) (e.g., document symptoms to discuss with providers); (III) Driving (*n* = 11) (e.g., discuss sleepiness and driving with primary care provider and PD specialist) and Falls (*n* = 10) (e.g., discuss neuropathy and falls with neurologist); and (IV) Financial/Community Resources (*n* = 9) (e.g., ask about transportation options with Home Health providers).

Of the activity, “Recommended a specific care intervention to participant” (*n* = 74) (Table 5), a total of 30 specific suggestions were available and abstracted. These were closed card sorted into two steps of the nurse care manager problem/topic-specific intervention protocol: (1) Providing information (e.g., chew gum to help swallow excess saliva for drooling problems, speak with Treatment Team before starting over-the-counter medications) and (2) Problem-solving collaboratively (e.g., manage pain issues first, wait 20–30 min after taking PD meds before shower/activities).

#### Nurse care manager coaching on the notebook

The initial nurse care manager Notebook-related activities were assessing receipt of Notebook, assessing Notebook use, and readiness to learn. The two Notebook coaching activities were: teaching about Notebook (*n* = 102 participants in 208 notes) and encouraging and complimenting participants about Notebook use (*n* = 87 participants in 176 notes). These activities were most frequently documented in Domain II Mental Status/Emotions/Coping (46.1%). A total of 69 unique education sheets were used by the nurse care managers. Each Notebook had 2.6 (standard deviation 2.4) additional education sheets based on the CHAPS Assessment findings and problem prioritization with the participant. These education sheets were distributed across all four domains.

#### Care coordination

Care coordination of referral recommendations involved 42 types of health care providers and services. The most frequent health referrals to providers (*n* = 233) were primary care providers, movement disorder specialists, and neurologists. The most frequent referrals to VA services (*n* = 190) were: (1) monthly PADRECC educational/supportive telephone conferences (PD at Home), (2) My HealtheVet, and (3) VA social work. Nurse care managers also recommended use of specific community services (*n* = 78). Care coordination through warm hand-offs (i.e., more immediate communication) most frequently involved PD specialists, care partners, primary care providers, and neurologists. These hand-offs were performed using live concurrent telephone discussions (*n* = 137), by co-signature of specific notes (*n* = 197), secure email (*n* = 20), and, occasionally, face-to-face discussions (*n* = 4).

### Participants’ concerns, self-care actions, and improvements

**Fig. 3:**
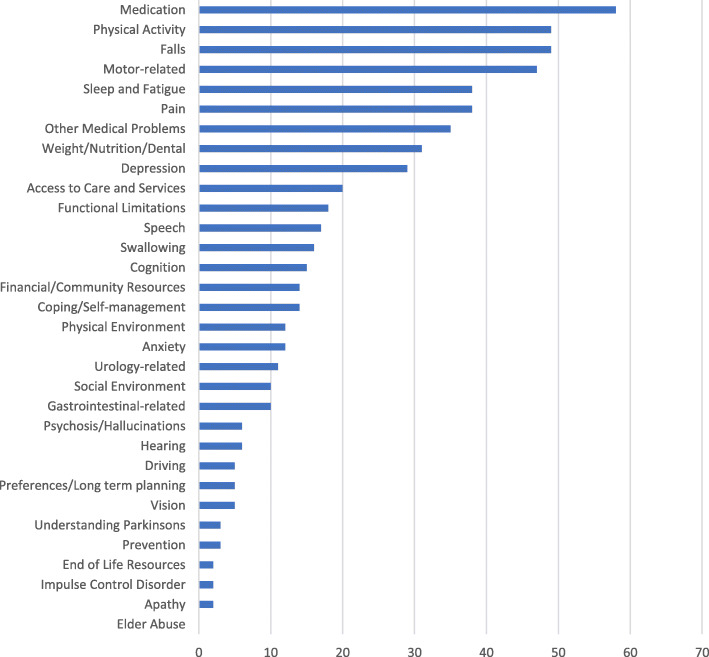
Frequency of unique participants’ concerns about CHAPS problems/topics and “Other Medical Problems”

Nurse care managers elicited participants’ concerns across multiple CHAPS problems/topics. The three most frequent concerns were about Medication, Physical activity, and Falls. Thirty-five participants had concerns about other medical problems (e.g., diabetes, hypertension, osteoarthritis) (Fig. 3).

Participants demonstrated self-care through multiple actions across all four domains. The most frequent were coping-focused: follow up with providers and interacting with the Notebook (Table 6). For unique participants with documentation about the Notebook (*n* = 112), the most frequent actions were reading Notebook text (*n* = 70, 62.5%), showing the Notebook to their care partner (*n* = 30, 26.8%), taking Notebook to a provider appointment (*n* = 18, 16.1%), and writing questions for providers (*n* = 16, 14.3%).

**Table 6:** Participants’ self-care action frequency documented by nurse care manager in notes and by unique participant

Participants’ self-care actions	Notes (n = 656) n (%)	Participants (n = 140) n (%)
I Medically-focused^a^		
Adhering to medications	129 (19.7)	63 (45.0)
Understands medication	82 (12.5)	43 (30.7)
Management weight/nutrition/diet component(s)	28 (4.3)	17 (12.1)
II Mentally/emotionally/coping-focused		
Made appointments with medical providers	330 (50.3)	108 (77.1)
Prepared/preparing for appointment with provider	201 (30.6)	82 (58.6)
Interacted with Siebens Health Care Notebook	144 (22.0)	83 (59.3)
Read specific nurse-supplied materials	54 (8.2)	37 (26.4)
Going to support group	34 (5.2)	20 (14.3)
Preparing/applying for benefits	31 (4.7)	14 (10.0)
Completing/completed advance directives/durable power of attorney for health care	30 (4.6)	21 (15.0)
Using My HealtheVet	26 (4.0)	12 (8.6)
Participating in Parkinson’s Disease at Home telephone call	13 (2.0)	7 (5.0)
Going to speech therapy	6 (0.9)	6 (4.3)
Attending Parkinson disease conferences	2 (0.3)	2 (1.4)
III Functionally-focused		
Doing volunteer/paid work/project/leisure activities	98 (14.9)	51 (36.4)
Exercising/physical therapy	97 (14.8)	57 (40.7)
Occupational therapy	1 (0.1)	1 (0.7)
IV Environmentally-focused		
Utilizing community resources (e.g., Veteran Service Organization, religious affiliations)	16 (2.4)	9 (6.4)
Utilizing community-based health resources	6 (0.9)	5 (3.6)
Utilizing adult day health care	2 (0.3)	1 (0.7)
Utilizing senior centers	3 (0.5)	3 (2.1)
Utilizing transportation services	2 (0.3)	1 (0.7)

^a^ Sections headings from the Siebens Domain Management Model™ used with permission

A total of 79 participants reported specific improvements they experienced, as documented by nurse care managers in 142 (21.7%) notes. The most frequently reported improvements were Motor-related (n = 19), Sleep and fatigue (n = 16), Weight/nutrition/dental (*n* = 15), Pain (n = 11), Swallowing (*n* = 10), Depressive symptomatology (n = 10), and Falls (n = 10).

### Decision-support activities

Nurse care managers had monthly huddles with site PD specialist champions to collaborate and review patient issues for decision-support and future planning. These occurred in person or by email/telephone. Nurse care managers held team conference calls among themselves to discuss participants’ challenging problems/topics and their care management.

## Discussion

Findings in this study demonstrated nurse care managers implemented the CHAPS intervention following the CHAPS protocol. Participants had multiple health issues and many received care from several sources, requiring care coordination. Nurse care managers identified participant’s individual learning preferences and health information tracking practices to facilitate coaching for a breadth of problems/topics in participants’ self-care.

Through the structured CHAPS Assessment, nurse care managers proactively and systematically identified a range in frequency of all 31 unique problems/topics that participants experienced. Few additional problems/topics were identified indicating the CHAPS Assessment was comprehensive in addressing PD-related health problems. The nurse care manager-identified problems overlapping with participant concerns were Medications, Falls, and Motor-related problems. Differences highlighted the importance of prioritizing problems/topics collaboratively. The multiplicity of problems justified standardized assessments to avoid missing risk factors (e.g., dysphagia, confusion, gait instability, unsafe home). Care management of these risks, before crises occur, may prevent worsening health and hospitalizations, a driver of annual costs [16].

Nurse care managers regularly used the Organizing Framework to document problems/topics identified during care management. These were distributed over all four domains, facilitating a person-centered perspective. As quality of life can be influenced by factors in any domain, assessing for concerns in all four domains was necessary (e.g., depressive symptoms, declining functional status, and social isolation) [24, 57–59].

Study results indicated that nurse care managers performed many unique activities. The most frequent was providing counseling and emotional support in addition to multiple other activities (e.g., medication management, verbal education, initiating care coordination). Medication management is especially important in individuals with PD who often experience multimorbidity and have multiple providers. Nurse care managers recommended topics to discuss with providers thereby teaching participants’ how to talk to doctors and others about their own care. Because PD care requires interaction with different providers and services [60, 61], the nurse care manager was an ideal single point person for care personalization, assessment-driven coaching, and care coordination.

From this analysis, evidence was gained concerning participants’ self-care actions, which occurred over a range of health concerns such as understanding medications, active coping (e.g., support groups), engaging in physical activities, and utilizing community resources. As part of coping, participants interacted with the Notebook [5, 49]. Those self-managing using the Notebook increased over time. Participants mentioned some personal health improvements, potentially indicating benefits from their self-care actions.

### Comparisons with recent literature

The design and delivery of the CHAPS program [30] were consistent with additional recent recommendations and research for improving PD care. As in ParkinsonNet, CHAPS included education of staff (nurse care managers), collaboration (huddles with PD specialists, warm hand-offs in care coordination), and following established care indicators and guidelines (PD quality indicators) [16, 19, 22]. Nurse care manager activities met the six minimum standard tasks for PD nurse specialists: provide information and education, support patient and caregiver in self-management, screen and offer prevention, support patient and caregivers on psychosocial and existential domains, work in multidisciplinary collaboration, and perform specific nursing-technical interventions [19]. CHAPS problems/topics, using the Organizing Framework, overlapped with the PD nurse specialist guideline areas: nutrition, sexuality, sleep (Domain I); mental functioning, palliative care (Domain II); and self-care, mobility and work (Domains III and IV) [19]. CHAPS addressed the challenge of limited numbers of PD nurse specialists by developing a concise CHAPS orientation program for nurses from different backgrounds. This study’s findings are consistent with other descriptions of intervention delivery characteristics [28, 29] except for examination of relationships among intervention characteristics and clinical outcomes [62].

### Limitations

Results may not be generalizable beyond this mostly male veteran sample and VA setting. Participants’ improvements could have resulted from events or treatments unrelated to CHAPS. The intervention’s implementation was less intense than initially planned across the 18-month intervention [22]. We were unable to perform further analyses to characterize (1) those terminated from the study (*n* = 17) after receipt of the initial assessment (*n* = 140) and (2) those who remained in the study yet did not receive the protocol-specified nurse care manager follow-up. However, the main reason for lack of follow-up was nurse care manager unavailability due to a hiring freeze, a reason unlikely to introduce bias. Some data collection was potentially incomplete as clinicians are often unable to document everything they do [63]. However, the CHAPS standardized Assessment structure and use of the Organizing Framework may have assisted in making the documentation more thorough. Despite these limitations, we believe data collected about the care management activities reflect the quality and extent of the intervention’s implementation.

CHAPS did not target specific acuity groups as is often recommended for effective care management [64]. However, the CHAPS standardized Assessment accommodated a range of PD severity. The Assessment’s structure included algorithms triggering a range of nurse care manager activities based on participant responses (e.g., mild drooling: Swallowing intervention protocol; moderate/severe drooling: protocol and referral to PD specialist).

### Implications

In examining the CHAPS intervention implementation, the protocol components were identified, confirming they occurred. Findings on implementation quality and extent support dissemination, adding to support from the previous positive findings from the randomized trial. Responses of stakeholders (participants, nurse care managers, and PD specialists) to the implementation are also required [21] and will be reported separately. Together these findings can inform decision-makers about CHAPS dissemination.

Key features of the CHAPS program may help facilitate its spread while considering costs. The CHAPS proactive care management elements are adaptable to addressing other enduring health conditions [5, 6, 65]. Also, proactive, organized, and standardized care management may allow for a more dependable care environment for patients, their providers, and administrators responsible for operations. While hospitalizations were not decreased in CHAPS, risk factors for hospitalization were identified and addressed that may contribute to future cost savings [22].

Several operational costs include nurses, the Organizing Framework, and the Notebooks. Current nursing staff at sites, if available, can learn to provide CHAPS as occurred during implementation. If sites do not have available nursing capacity, nursing staff would need to be hired. The research findings may help overcome resistance to hiring nurses. Additionally, a qualitative analysis of CHAPS stakeholder (i.e., participant, nurse care manager, PD specialist) perceptions, to be reported separately, can inform cost decisions. These resources are designed to accommodate other patient conditions (i.e., spreading the cost) and improve communication (i.e., less wasted time) within and outside health care organizations, strengthening care management practices for enduring health conditions in feasible ways [17]. A formal analysis of whether the CHAPS intervention was cost-effective was outside the scope of our study. However, CHAPS research findings demonstrated that CHAPS likely represented reasonable value for money spent. Therefore, CHAPS may be a real-world viable approach to improve care quality of individuals with a variety of enduring health conditions.

Given attention to PD quality indicators in CHAPS’ design, the intervention provides a means to decrease practice variation across nurse care managers. Insights from study findings may help develop a nursing care coordination quality measure [66]. Also, CHAPS may benefit some participants when adapted to clinical video telehealth in either rural or urban environments [67–69]. Further, nurse care manager assessment or review of tools like My HealtheVet and the Notebook may be more appropriate done in person for some individuals and help establish the nurse care manager/participant relationship.

Despite the clear devotion of the nurse care managers to the participants and improvements in care processes, it is of interest that additional secondary outcomes in the CHAPS randomized controlled trial [22] did not differentially improve in intervention versus control, based on intention-to-treat, except for the screen for depression symptoms. This may have been related to: (1) the choice of patient-reported outcomes for this first CHAPS trial, (2) the study sample size in relation to these specific outcomes, (3) nurse care managers needing to learn a new structured approach to PD care, (4) limitations in CHAPS design itself (e.g., software, design of initial nurse care manager education and practice), and (5) care management intensity that was limited as not all participants received the Assessments or all planned monitoring and follow-up.

## Conclusions

CHAPS nurse care managers implemented multiple activities including participant coaching and care coordination per the CHAPS protocol. Participants reported various self-care actions including use of a personalized notebook. These findings indicate good quality and extent of the implementation, contribute to ensuring reproducibility, and support CHAPS dissemination as a real-world viable approach to improve care quality.

## Data Availability

By the time we deidentify data to degree that would be acceptable, too many key covariates are taken out, given veterans can be re-identified with enough social and/or personal demographic and area information. Thus, data are not available in a clinical trial database.

## Abbreviations

CHAPS: Care Coordination for Health Promotion and Activities in Parkinson’s Disease
PD: Parkinson disease
VA: Veterans Health Administration
FTEE: full time employee equivalent
PADRECC: Parkinson’s Disease Research, Education, and Clinical Centers
REDCap: Research Electronic Data Capture
SAS: statistical analysis software
